# Transcriptome analysis reveals similarities between human blood CD3^−^ CD56^bright^ cells and mouse CD127^+^ innate lymphoid cells

**DOI:** 10.1038/s41598-017-03256-0

**Published:** 2017-06-14

**Authors:** David S. J. Allan, Ana Sofia Cerdeira, Anuisa Ranjan, Christina L. Kirkham, Oscar A. Aguilar, Miho Tanaka, Richard W. Childs, Cynthia E. Dunbar, Jack L. Strominger, Hernan D. Kopcow, James R. Carlyle

**Affiliations:** 1 Department of Immunology, University of Toronto, and Sunnybrook Research Institute, Toronto, Ontario, Canada; 2 Hematology Branch, National Heart, Lung, and Blood Institute, National Institutes of Health, Bethesda, Maryland USA; 3 Beth Israel Deaconess Medical Center, Harvard Medical School, Boston, Massachusetts, USA; 4 Department of Stem Cell and Regenerative Biology, Harvard University, Cambridge, Massachusetts, USA; 5 Nuffield Department of Obstetrics & Gynaecology, University of Oxford, Oxford, UK

## Abstract

For many years, human peripheral blood natural killer (NK) cells have been divided into functionally distinct CD3^−^ CD56^bright^ CD16^−^ and CD3^−^ CD56^dim^ CD16^+^ subsets. Recently, several groups of innate lymphoid cells (ILC), distinct from NK cells in development and function, have been defined in mouse. A signature of genes present in mouse ILC except NK cells, defined by Immunological Genome Project studies, is significantly over-represented in human CD56^bright^ cells, by gene set enrichment analysis. Conversely, the signature genes of mouse NK cells are enriched in human CD56^dim^ cells. Correlations are based upon large differences in expression of a few key genes. CD56^bright^ cells show preferential expression of ILC-associated *IL7R* (CD127), *TNFSF10* (TRAIL), *KIT* (CD117), *IL2RA* (CD25), CD27, *CXCR3, DPP4* (CD26), *GPR183*, and MHC class II transcripts and proteins. This could indicate an ontological relationship between human CD56^bright^ cells and mouse CD127^+^ ILC, or conserved networks of transcriptional regulation. In line with the latter hypothesis, among transcription factors known to impact ILC or NK cell development, *GATA3*, *TCF7* (TCF-1), *AHR*, *SOX4, RUNX2*, and *ZEB1* transcript levels are higher in CD56^bright^ cells, while *IKZF3* (AIOLOS), *TBX21* (T-bet), *NFIL3* (E4BP4), *ZEB2*, *PRDM1* (BLIMP1), and *RORA* mRNA levels are higher in CD56^dim^ cells.

## Introduction

Natural killer (NK) cells are an important component of the innate immune system that serve the dual functions of direct cellular cytotoxicity and early secretion of regulatory cytokines. Phenotypically, human NK cells have been distinguished by their expression of CD56 (NCAM1), and the absence of CD3. For almost 30 years, human NK cells have been further classified into two sub-populations based upon surface levels of CD56 and CD16 (FCGR3A)^1, 2^. The first population, composed of CD56^bright^ cells, make up approximately 10% of circulating blood NK cells, and are characterized by high-density expression of CD56 and low or negative levels of CD16. The second population, CD56^dim^ cells, make up the remaining ~90% of blood NK cells, and are characterized by low-density expression of CD56 and high levels of CD16. These two populations show distinguishing differences in expression of inhibitory NK cell receptors, cytokine and chemokine receptors, as well as differential functional responses (reviewed in ref. 2). For instance, CD56^dim^ cells exert greater cytotoxic effects on target cells^1^, but produce lesser quantities of cytokines^3^. On the other hand, *ex vivo* CD56^bright^ cells are weakly cytotoxic^1^, and produce high levels of immunoregulatory cytokines, such as IFN-γ, lymphotoxin-α and GM-CSF^3^.

The developmental relationship of the CD56^bright^ and CD56^dim^ populations remains unclear. Some reports have provided evidence that CD56^bright^ NK cells may be developmental precursors of CD56^dim^ NK cells^4–6^. Upon *in vitro* culture with synovial fibroblasts or cytokines, CD56^bright^ NK cells were reported to undergo multiple changes in cell surface phenotype and function to resemble CD56^dim^ NK cells^4, 6^. Another study observed acquisition of CD16 on sorted CD56^bright^ cells, but not other features of CD56^dim^ cells, upon culture in IL-15; this process could be regulated by TGF-β^7^. CD56^bright^ cells were shown to have longer telomeres than CD56^dim^ cells, perhaps consistent with a more immature status^4, 6^. On the other hand, studies following clonal hematopoiesis *in vivo* in rhesus macaques revealed that analogous CD56^+^CD16^−^ and CD16^+^ NK cell populations showed differences in progenitor cell origin for many months after stem cell transplant; a large number of rhesus CD16^+^ NK cells were derived from highly biased progenitor clones that did not substantially give rise to other lineages, while many CD56^+^CD16^−^ cells shared progenitors with T, B, and myeloid cells^8^. This provides evidence that human CD56^bright^ and CD56^dim^ populations may follow distinct developmental pathways. Interestingly, some patients deficient in GATA2 lack CD56^bright^ blood cells, while they retain some CD56^dim^ NK cells, possibly arguing against a simple precursor-progeny relationship^9^. Mouse NK cells do not express CD56, making assignment of analogous mouse populations more challenging.

In the past few years, a number of related subsets of innate lymphoid cells (ILC), distinct from NK cells, have been described in mouse and human. These include: (i) Rorγt-dependent group-3 ILC (ILC3), which produce IL-22 and play key roles in bacterial and fungal defence, mucosal homeostasis, regulation of immune tissue development, and modulation of adaptive immune responses; (ii) group-2 ILC (ILC2), expressing high levels of GATA3, which produce Th2-associated cytokines, including IL-5 and IL-13, and profoundly impact allergic responses and parasite defence; and (iii) T-bet (*Tbx21*)-dependent, group-1 ILC (ILC1) cells, which produce IFN-γ and are reported to be distinct from NK cells in development and function (reviewed in ref. 10). Using fluorescent *Id2* or *Zbtb16* (PLZF) reporter mice, along with surface marker staining, two groups defined common lymphoid progenitor (CLP)-like precursor cells with the capacity to differentiate *in vivo* or *in vitro* into ILC3, ILC2, and ILC1 cells, but not conventional mouse NK cells^11, 12^. The developmentally distinct mouse ILC1 subset appears to include TRAIL^+^ (*Tnfsf10* ^+^) DX5^−^ (CD49b^−^) CD49a^+^ NK1.1^+^ liver cells, previously described to display an immature NK phenotype^13^ or termed tissue resident NK cells^14–16^. Genetic lineage-tracing/fate-mapping studies showed that most liver NK1.1^+^ DX5^−^ ILC1 cells had developed via a PLZF^+^ precursor, while most NK1.1^+^ DX5^+^ liver and spleen NK cells had not, supporting different developmental pathways^11^. In mice, Eomes is reported to distinguish conventional NK cells from ILC1, with lack of Eomes correlating with a TRAIL^+^ DX5^−^ phenotype in the liver^17, 18^ and CD127^+^ CD27^+^ phenotype in the small intestine^12^. Both Eomes^+^ NK cells and Eomes^−^ ILC populations can express the typical mouse NK cell markers, NK1.1, NKp46, and NKG2D, complicating their separation^12, 17, 18^. It has been proposed that all “helper ILC” (ILC3, ILC2, and ILC1) express CD127 (*Il7r*), implying that ILC1 may be distinguished from NK cells by CD127 expression. However, both ILC1 and NK cells depend mainly upon IL-15 for their development (rather than IL-7), and both subsets develop in thymectomized and *Foxn1* ^−/−^ mice^12, 15, 17^. Genetic lineage-tracing experiments also suggest that mouse Rorγt^+^ ILC3 can lose Rorγt expression to acquire an ILC1-like phenotype, producing IFN-γ and expressing NK1.1 and NKp46^12, 19^; these transitioned cells have been referred to as “ex-ILC3 cells.” In human tonsil, non-NK ILC1 cells have been identified with a CD56^−^ CD127^+^ NKp44^−^ CD117^−^ perforin^−^ CRTH2^−^ phenotype^20^.

In light of the discovery of these varied ILC subsets, the nature of the human peripheral blood CD3^−^ CD56^bright^ subset is re-examined here bioinformatically, using transcriptome-wide expression data. Transcription factors distinguishing this subset are also identified.

## Results

### Signature transcripts of mouse ILC are enriched in human CD56^bright^ cells, while mouse NK signature gene products are enriched in human CD56^dim^ NK cells

**Figure 1:**
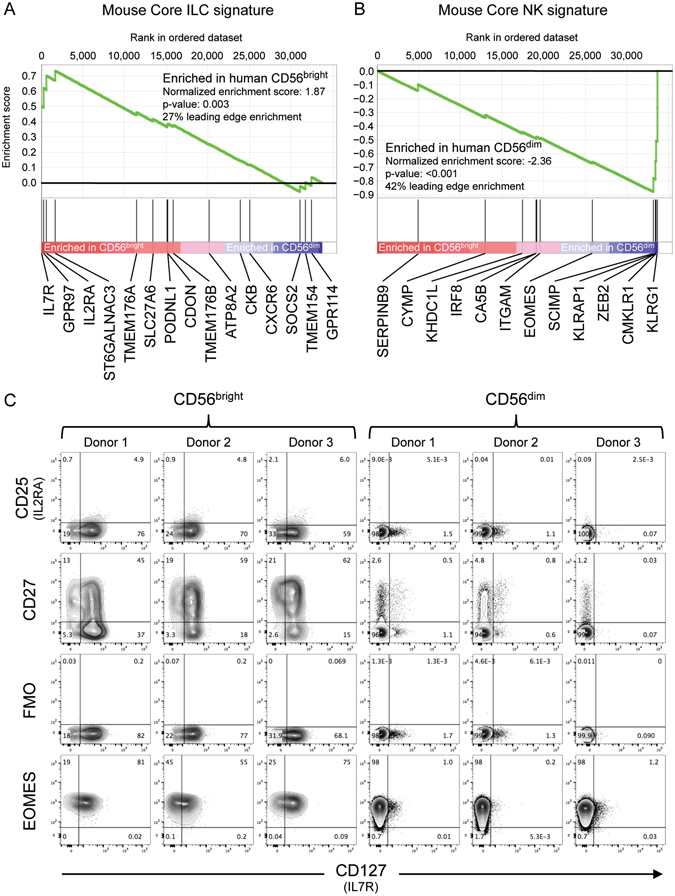
Signature genes, higher in mouse ILC subsets, are enriched in human blood CD3^−^ CD56^bright^ cells, while mouse NK cell signature genes are enriched in human CD3^−^ CD56^dim^ cells. (**A,B**) Transcriptional signatures of mouse NK cells (*Core NK*) and other mouse ILC (*Core ILC*) were defined by Robinette and colleagues as part of the ImmGen project^23^. Enrichment of corresponding homologous human genes was examined by Gene Set Enrichment Analysis in human blood CD56^bright^ CD16^−^ CD3^−^ and CD56^dim^ CD16^+^ CD3^−^ populations characterized by Affymetrix HTA2.0 microarray^21^. Deviations of cumulative scores (green line) above zero indicate enrichment in CD56^bright^ cells, while negative scores indicate enrichment in CD56^dim^ cells. Samples of each subset from three individuals were used for analysis. Signature genes without human homologues were not included in the analysis. (**C**) Flow cytometry staining of gated human blood CD56^bright^ CD16^−^ CD3^−^ or CD56^dim^ CD16^+^ CD3^−^ populations from three donors. Quadrant gates were set using fluorescence minus one (FMO) stains, of which one is shown. Intracellular staining for EOMES was performed on three blood samples distinct from those used to acquire the other results.

We undertook a re-evaluation of the nature of the human blood CD56^bright^ and CD56^dim^ cell subsets. Microarray results from an Affymetrix HTA2.0 dataset^21^ was used to compare peripheral blood CD3^−^ CD56^bright^ CD16^−^ and CD3^−^ CD56^dim^ CD16^+^ cells, with results confirmed, where indicated, with data from an independent microarray dataset based upon HG-U133 A and B arrays^22^. Robinette and colleagues previously examined multiple mouse ILC lineages by microarray in the context of the Immunological Genome Project (ImmGen), and used these data to define expression signatures: *Core ILC* signature genes were expressed at higher levels in multiple mouse ILC subsets, but were lower in conventional NK cells^23^; *Core NK* signature transcripts were selectively over-expressed in mouse NK cells^23^. After conversion of these signatures to homologous human genes, selective enrichment was examined in human CD56^bright^ and CD56^dim^ subsets using the Gene Set Enrichment Analysis (GSEA) algorithm (which calculates scores based on positions of signature genes within a rank-ordered list according to differential expression^24^; Fig. 1A,B). Interestingly, the mouse *Core ILC* signature was significantly enriched in human CD56^bright^ cells (p = 0.003) (Fig. 1A). However, this enrichment was largely driven by a few key signature genes that displayed expression highly biased towards CD56^bright^ cells. Comparing CD56^bright^ with CD56^dim^ cells, *IL7R* (CD127) was 22.9-fold higher; *GPR97*, 2.6-fold; and *IL2RA* (CD25), 1.6-fold. Independent human microarray data, from the second microarray platform, confirmed enrichment of the *Core ILC* signature in CD56^bright^ cells due, in part, to higher expression of *IL7R* and *GPR97* (an orphan G protein-coupled receptor) (Suppl. Figure 1A). In mouse, it has been proposed that surface CD127 (*Il7r*) distinguishes helper ILC lineages (ILC3, ILC2, and ILC1) from conventional NK cells. Strikingly, *IL7R* represented the transcript with the second highest fold-change between human CD56^bright^ and CD56^dim^ subsets, greater than CD16 (*FCGR3A*, 15.4-fold) and CD56 (*NCAM1*, 1.8-fold), the defining phenotypic markers of the subsets. At the protein level, CD127 appears to be bimodal on the surface of CD56^bright^ cells (Fig. 1C), perhaps indicating the presence of distinct subsets of cells within the CD56^bright^ population, or down-regulation upon recognition of IL-7. CD127 expression on CD56^bright^ cells has been reported previously^25^. CD56^dim^ cells appeared to be mostly negative for CD127 (Fig. 1C). Some CD56^bright^ cells also show low-level cell surface expression of CD25 (IL2RA) (Fig. 1C) as noted previously^26, 27^. Although not part of the bioinformatically-defined signature, CD27 is another marker that Klose and colleagues have reported to distinguish mouse ILC1 from conventional NK cells and ex-ILC3^12^. Interestingly, CD27 is also selectively expressed on the cell surface of CD56^bright^ cells in a bimodal fashion (Fig. 1C), again highlighting the possibility of distinct subsets. However, CD56^bright^ cells did not display substantially biased expression of other characteristic mouse ILC genes such as *CXCR6* (Fig. 1A).

Conversely, mouse *Core NK* signature genes were highly enriched in the human CD56^dim^ subset (p < 0.001) (Fig. 1B). This was largely driven by 5 genes within the signature: *KLRG1* (3.7-fold higher in CD56^dim^), *CMKLR1* (3.1-fold), *ZEB2* (2.7-fold), *KLRAP1* (1.8-fold), and *SCIMP* (1.5-fold). *KLRAP1* is the single human (pseudogene) homologue of the mouse Ly49 receptors, several of which are part of the mouse *Core NK* signature. Functional equivalents of the Ly49 in human, the killer-cell immunoglobulin-like receptors (KIR), are also highly differentially expressed between the human subsets. Twelve of thirteen KIR genes that were interrogated displayed extremely biased expression in CD56^dim^ cells, ranging from 3.2-fold to 13.4-fold higher in CD56^dim^ cells (the exception being KIR2DL4, which was similar in both subsets) (data not shown). Using mAb recognizing several KIR, selective staining of CD56^dim^ NK cells has been reported for many years^7, 28^. Examination of the second human microarray dataset also showed an enrichment trend of the mouse *Core NK* signature genes in CD56^dim^ cells (Suppl. Figure 1B). Reports have defined the transcription factor, Eomes, as critical in distinguishing mouse NK cells (which express Eomes) from ILC1 (which do not)^12, 17^. However, the human CD56^bright^ and CD56^dim^ subsets do not differ significantly in transcript levels of *EOMES* (measured on both microarray platforms). Similarly, intracellular flow cytometry staining revealed high levels of EOMES protein in blood CD56^bright^ and CD56^dim^ cells^29^, irrespective of CD127 expression (Fig. 1C).

### Transcripts varying between mouse splenic CD127^+^ ILC and CD127^−^ NK cells show commonalities with differential transcripts observed between human blood CD56^bright^ and CD56^dim^ subsets

**Figure 2:**
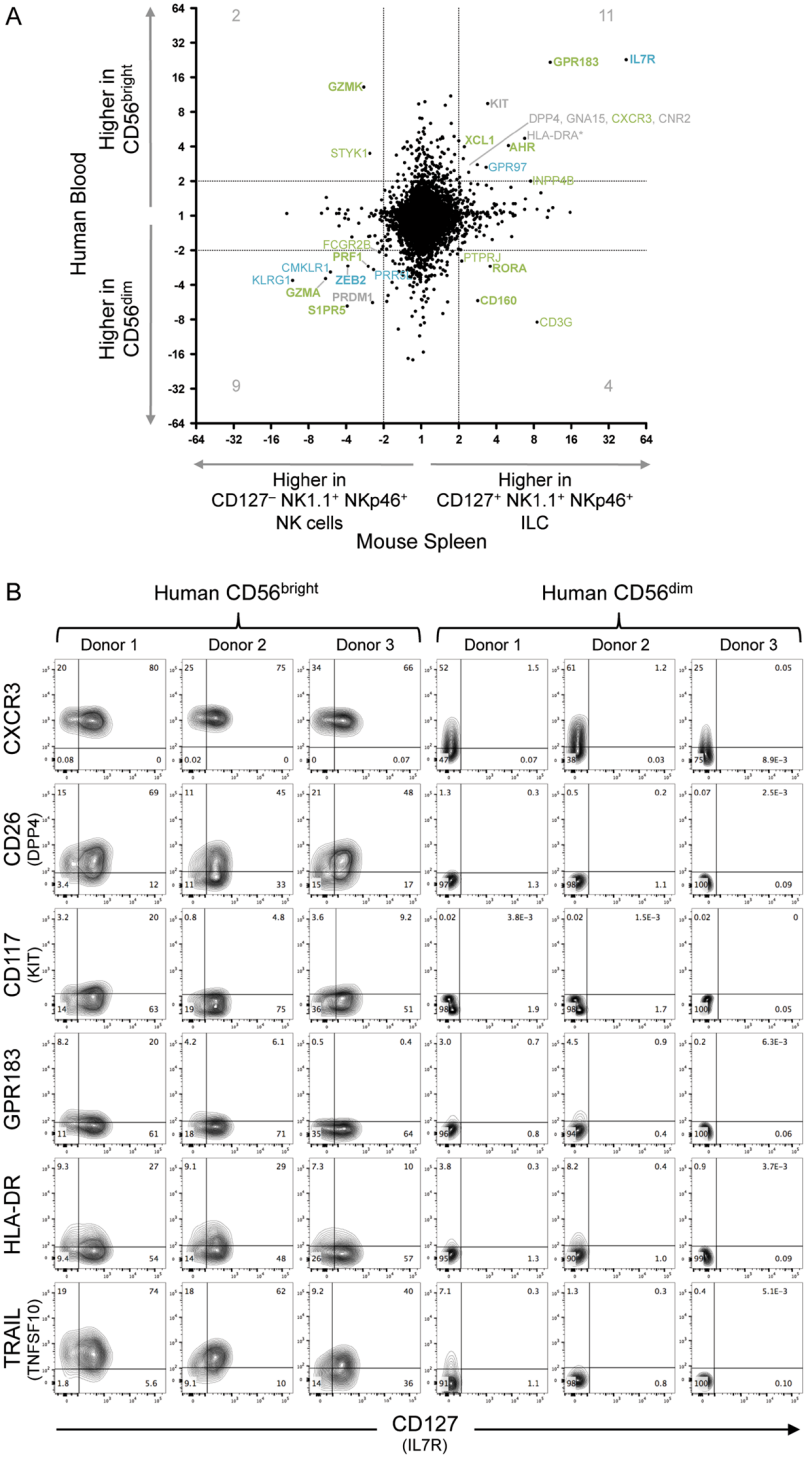
Expression differences between mouse splenic CD127^+^ NK1.1^+^ NKp46^+^ ILC and CD127^−^ NK1.1^+^ NKp46^+^ NK cells show commonalities with differences between human blood CD56^bright^ and CD56^dim^ cells. ImmGen microarray data examining mouse splenic NK1.1^+^ NKp46^+^ CD127^+^ CD27^+^ cells (annotated as ILC1) and NK1.1^+^ NKp46^+^ CD127^−^ CD27^+/−^ NK cells^23^ is analysed to show fold-change difference between subsets (x-axis). For all mouse genes with corresponding human genes in the NCBI Homologene database, this is contrasted with microarray expression differences observed between human blood CD56^bright^ and CD56^dim^ populations (y-axis) (HTA2.0 microarray)^21^. Transcripts differing by ≥2-fold between both human and mouse subsets are labelled, and their numbers are shown. Transcripts similarly varying by ≥2-fold in mouse ILC1/NK comparisons in both liver and small intestine (in addition to spleen) are shown in blue; those differing by ≥2-fold in 2-of-3 comparisons of mouse tissues are in green (see Suppl. Figure 3). Bold names also show ≥2-fold differences in an independent microarray dataset comparing human CD56^bright^ and CD56^dim^ cells (see Suppl. Figure 4). Although not matched by Homologene, human HLA-DRA (paired with mouse H2-Aa) is shown (with asterisk) but not included in transcript counts. (**B**) Flow cytometry staining of gated human blood CD56^bright^ CD16^−^ CD3^−^ or CD56^dim^ CD16^+^ CD3^−^ populations from three donors. Quadrant gates were set using fluorescence minus one (FMO) stains.

In order to more closely examine these similarities, we examined the overall correlation of gene expression differences between the human blood subsets with differences between mouse ILC and NK cell subsets. Robinette and colleagues examined ILC and NK cell populations in several mouse tissues by microarray. In spleen, ILC1 were isolated as NK1.1^+^ NKp46^+^ CD127^+^ CD27^+^, while NK cells were NK1.1^+^ NKp46^+^ CD127^−^ (CD27^+/−^)^23^; thus CD127 and to some extent CD27 were the defining differential markers. To compare across species, homologues were matched using the NCBI homologene database. This allowed analysis of 16,351 transcripts between human and mouse. Among these transcripts, 355 were found to differ by ≥2-fold in expression between human blood CD56^bright^ and CD56^dim^ subsets. Similarly, 184 differed by ≥2-fold between mouse splenic ILC1 and NK cells. Only 26 gene products overlapped between these two comparisons, showing that most differences between the subsets were species-specific. However, 20 of these 26 overlapping gene products (77%) differed in the same direction; that is, higher together in ILC1 and CD56^bright^ cells or higher together in mouse NK and CD56^dim^ cells. This is illustrated in Fig. 2A in the larger number of gene products in the upper right and bottom left, compared with the other two quadrants (p = 0.02 when compared by Fisher’s exact test). Although the overall relationship was not monotonic (Fig. 2A), Spearman correlation analysis was performed considering only the subset of gene products that differed ≥2-fold in expression between mouse splenic ILC1 and NK cells (p = 0.0005). However, the correlation coefficient was low (r = 0.25). Examined a different way, using the GSEA algorithm, mouse genes that were ≥2-fold higher in spleen CD127^+^ ILC1 showed significantly enriched expression in human CD56^bright^ cells (p < 0.001), while mouse genes ≥2-fold higher in spleen NK showed enrichment within the human CD56^dim^ population (p < 0.001) (Suppl. Figure 2). However, it is visually evident that this was due to a limited number of key transcripts (Fig. 2A).

Mouse liver and small intestine ILC1/NK cell subsets were also part of the ImmGen analysis, although isolated in a slightly different fashion. Again, both subsets had been sorted NK1.1^+^ NKp46^+^, but liver ILC1 were isolated as DX5^−^ TRAIL^+^, while NK cells were DX5^+^ TRAIL^− ^^23^. In the small intestine lamina propria, ILC1 were separated as RORγt^−^ CD127^+^, while NK cells were RORγt^−^ CD127^− ^^23^. A Rorγt^EGFP^ reporter had been utilized to exclude ILC3, which are predominant in intestinal tissue. No significant associations were found when similar Fisher’s exact test or Spearman correlation analyses were performed comparing human CD56^bright^/CD56^dim^ subsets with mouse ILC1/NK cells from either liver or small intestine (Suppl. Figure 3 **)**. However, some genes displayed similar patterns of expression in multiple tissues. Two genes were expressed ≥2-fold higher in human CD56^bright^ cells and mouse ILC1 subsets from all three tissues examined (*IL7R* and *GPR97*, both part of the *ILC Core* signature; see blue labels, Fig. 2A, Suppl. Figure 3). Five additional genes showed levels ≥2-fold higher in CD56^bright^ cells and ILC1 subsets from two of the mouse tissues (*GPR183*, *AHR*, *CXCR3*, *XCL1*, and *INPP4B*; green labels, Fig. 2A, Suppl. Figure 3). In the opposite direction, four genes showed higher levels in CD56^dim^ NK cells and mouse NK subsets from all three tissues (*KLRG1*, *ZEB2*, *PRR5L*, and *CMKLR1*; blue labels, Fig. 2A, Suppl. Figure 3). Seven were higher in CD56^dim^ cells and NK subsets from two of the mouse tissues (*S1PR5*, *GZMA*, *PRF1*, *FCGR2B*, *SLAMF6*, *ANXA2*, *GNPTAB*; green labels, Fig. 2A, Suppl. Figure 3). If the stringency is lessened to a ≥1.5-fold difference, *LTB*, *DTX1*, *CAMK4*, *GPR34*, and *IL2RA* transcript levels are higher in CD56^bright^ cells and mouse ILC1 subsets from all three tissues, while *RAP1GAP2*, *NDRG1*, *KLF12*, and *SCIMP* are higher in CD56^dim^ cells and all three mouse tissue NK subsets (data not shown). Similar analyses were performed using the second independent microarray dataset comparing human blood CD56^bright^ and CD56^dim^ subsets and revealed a similar pattern and confirmed many of the differentially expressed genes (Suppl. Figure 4). Interestingly, *LTB* (Lymphotoxin β) and *TNFSF10* (TRAIL) were preferentially expressed in CD56^bright^ cells in this analysis (Suppl. Figure 4). For a number of the genes that showed selective expression in mouse ILC1, cell surface protein was confirmed on human CD56^bright^ cells (Fig. 2B). CD56^bright^ cells showed considerable staining with monoclonal antibodies recognizing CXCR3, CD26 (DPP4), and TRAIL (TNFSF10) plus some reactivity with anti-CD117 (KIT) and anti-GPR183; in each case, staining of CD56^dim^ cells was lower or negative (Fig. 2B). Some of these observations have also been reported by others^30–33^. TRAIL expression is particularly striking, since TRAIL is used as a defining marker of ILC1 in mouse.

Although not necessarily matched between species by homologene, it is interesting to note that some MHC class II molecules were higher in both mouse splenic ILC1 and human CD56^bright^ cells (Fig. 2A). Expression of HLA-DR surface protein on some CD56^bright^ cells, but not CD56^dim^ cells, was observed (Fig. 2B)^25^ as has selective expression of HLA-DR on human ILC3^34^. Transcript levels of *KLRB1* (CD161, NKR-P1A) were comparable between human blood subsets (1.3 fold higher in CD56^dim^).

It has been reported that some ILC3 can exhibit lineage-plasticity, losing Rorγt expression and acquiring a phenotype similar to ILC1; the resulting cells are called “ex-ILC3”^12, 19^. Therefore, the mouse cells isolated as CD127^+^ NK1.1^+^ NKp46^+^ (and termed “ILC1” above) may also may also encompass or include these ex-ILC3 populations. Thus, it is possible that the human CD56^bright^ cell transcriptome (or subset(s) therein) may show commonalities with either mouse ILC1 or ex-ILC3 populations.

### Human CD56^bright^ cells express higher levels of some transcripts that are also selectively expressed in mouse ILC1 and ex-ILC3

**Figure 3:**
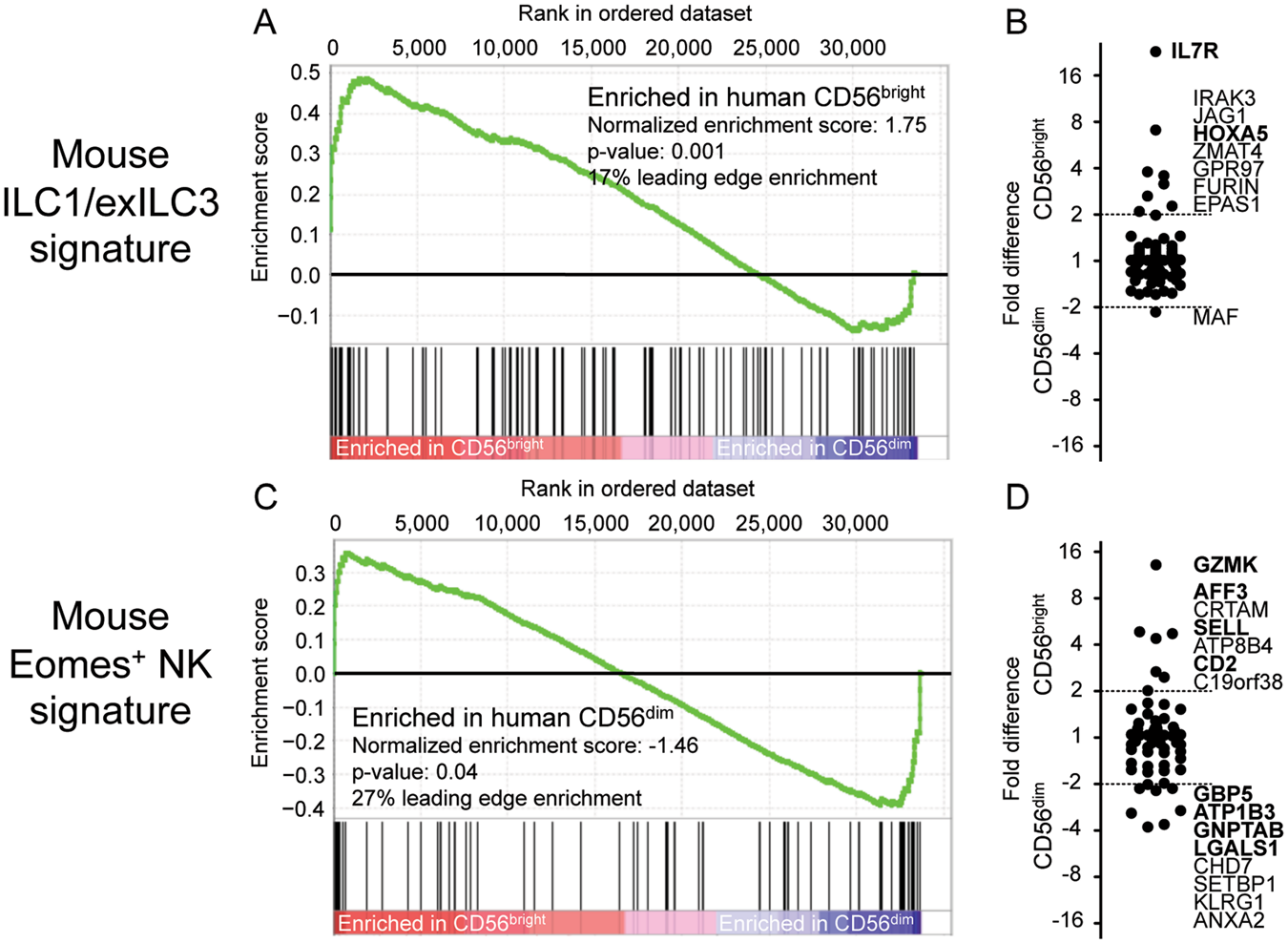
Transcripts with higher expression in mouse ILC1 and ex-ILC3 subsets show enrichment in human CD56^bright^ cells. Lists of signature transcripts were compiled from published microarray data comparing three mouse NK1.1^+^ NKp46^+^ subsets: (i) Eomes^+^ NK cells, (ii) Rorγt^fate map+^ ex-ILC3 (that formerly expressed Rorγt), and (iii) ILC1 that expressed neither (from ref. 12). Enrichment of corresponding homologous human genes was examined by Gene Set Enrichment Analysis in human CD56^bright^ and CD56^dim^ populations characterized by Affymetrix HTA2.0 microarray^21^, using the following signatures: (**A-B**) *Mouse ILC1/ex-ILC3 signature* (transcripts detected at ≥2-fold higher levels in *both* ILC1 versus NK and ex-ILC3 versus NK comparisons and (**C-D**) *Mouse Eomes* ^+^ *NK cell signature* (genes with ≥2-fold higher expression in *both* NK versus ILC1 and NK versus ex-ILC3 comparisons). (**B,D**) Fold difference in expression of these transcripts between human CD56^bright^ and CD56^dim^ cells is shown for comparison. Genes differing by ≥2-fold between the human subsets are labelled (in order by fold change difference) and emboldened if conserved in similar analyses with a second human microarray dataset (see Suppl. Figure 5).

Klose and colleagues employed a different strategy to examine the diversity of mouse NK1.1^+^ NKp46^+^ cells, utilizing a dual-reporter mouse. A heterozygous knock-in of GFP at the *Eomes* locus (*Eomes* ^GFP/+^) reported Eomes expression, while transgenic *Rorc*(γt)-Cre x *Rosa26R* ^Yfp/+^ provided fate mapping for prior Rorγt expression characteristic of ILC3^12^. The authors used this system to define three NK1.1^+^ NKp46^+^ populations as follows: (i) Eomes^+^ classical NK cells, (ii) Rorγt^fate-map+^ ex-ILC3, and (iii) ILC1, which expressed neither^12^ (with the minor caveats of monoallelic reporter expression and inappropriate transgene expression possible). Using microarray data characterizing these three mouse populations from small intestine^12^, we created lists of signature genes selectively expressed in the different subsets. These were then contrasted with human CD56^bright^ and CD56^dim^ cells by GSEA. Mouse transcripts enriched ≥2-fold in *both* ILC1 and ex-ILC3 relative to NK cells (*mouse ILC1/ex-ILC3 signature*) were significantly over-represented in the CD56^bright^ population (Fig. 3A). This was due to higher expression of several signature genes, including *IL7R*, *GPR97, HOXA5, IRAK3*, *ZMAT4*, and *EPAS1* in human CD56^bright^ cells (Fig. 3B), all of which were also identified by previous analyses in either Fig. 2A or Suppl. Figure 3 using ImmGen data. A similar result was observed using the other human microarray dataset (Suppl. Figure 5A,B). On the other hand, signature transcripts of mouse Eomes^+^ NK cells (≥2-fold higher in both NK versus ILC1 and NK versus ex-ILC3 comparisons) were reported by the GSEA algorithm to be enriched in human CD56^dim^ cells (Fig. 3C). However, inspection of the GSEA plot shows that the Eomes^+^ NK signature transcripts are actually preferentially clustered at both ends of the scale, some enriched in the CD56^bright^ and some in the CD56^dim^ subsets (Fig. 3C,D, Suppl. Figure 5C,D). Signature transcripts selectively found in only Rorγt^fate-map+^ ex-ILC3 appeared to be enriched in expression in human CD56^bright^ cells; however, this was mostly due to expression of one gene, *GPR183*, shared by both CD56^bright^ cells and mouse ex-ILC3 (Suppl. Figure 6A,B,E,F). *GPR183* (EBI2), a G-protein-coupled migration receptor recognizing oxysterols^35^ also features prominently in the Fig. 2A analysis using ImmGen data described above, although cell surface expression of GPR183 on CD56^bright^ cells appeared to be quite low (Fig. 2B). Enrichment patterns of mouse ILC1 signature transcripts were inconsistent between the two human microarray datasets (Suppl. Figure 6C,D,G,H). To gauge the magnitude of the GSEA enrichments that were observed here, the same mouse ILC signatures were also compared by GSEA with the other mouse microarray dataset from ImmGen (Suppl. Figure 7).

In summary, human CD56^bright^ cells shared the selective expression of a limited subset of genes with mouse spleen CD127^+^ NK1.1^+^ NKp46^+^ ILC and with ILC defined by other reporters. Two hypothetical interpretations of the preceding data include:

- Ontological/developmental/lineage similarity of CD127^+^ human blood CD56^bright^ cells with mouse spleen CD127^+^ NK1.1^+^ NKp46^+^ ILC, or
- Presence in both cell types of a similar module of gene regulation, perhaps due to expression of particular transcription factors.

To investigate the latter possibility, transcription factor expression in CD56^bright^ and CD56^dim^ cells was examined in greater detail.

### Human CD56^bright^ and CD56^dim^ cells differ in expression of multiple transcription factors previously linked to ILC and NK cell development

**Figure 4:**
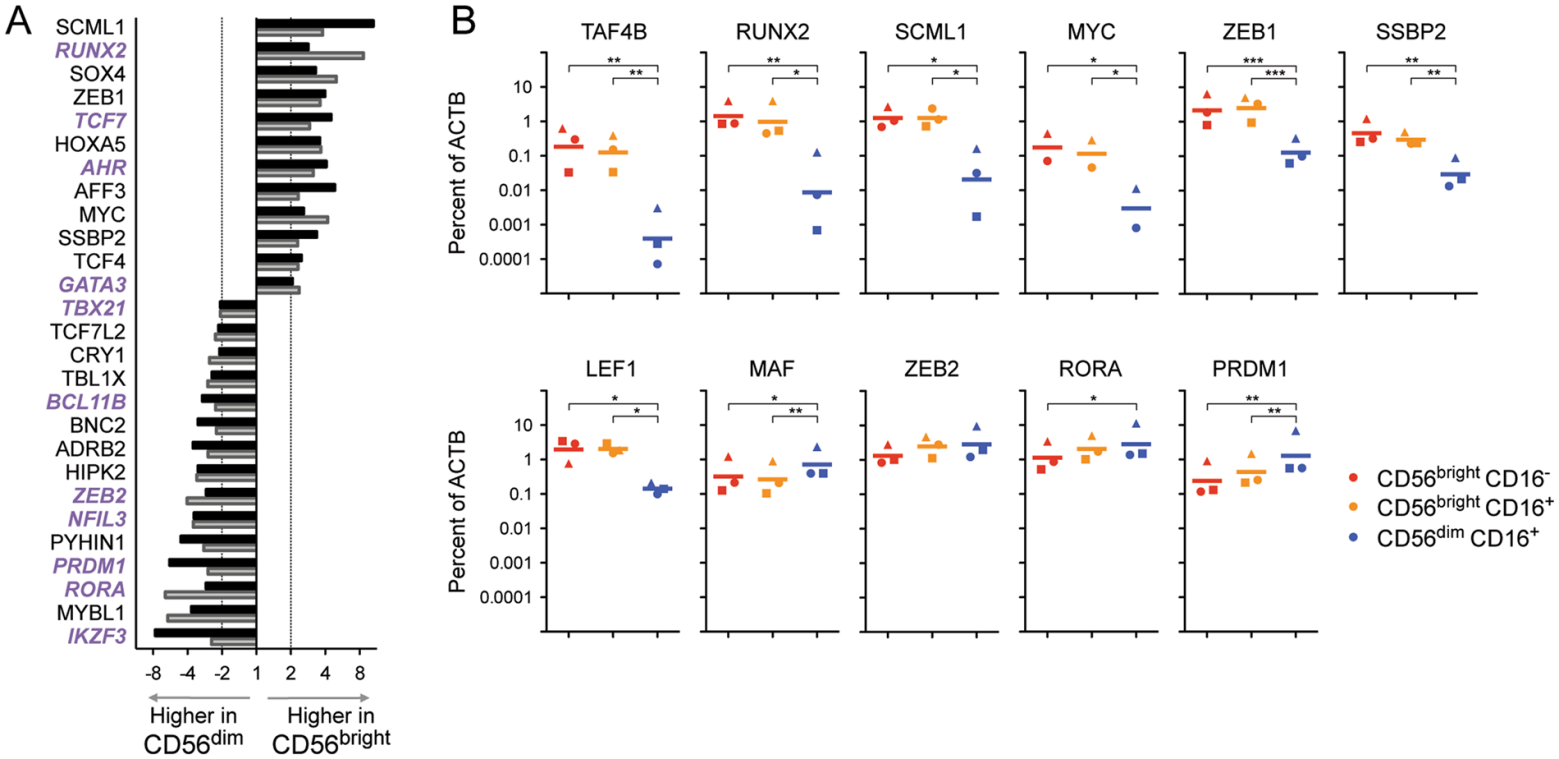
Human CD56^bright^ and CD56^dim^ subsets differ in expression of many transcription factors known to affect development of ILC and NK cells. (**A**) Fold-change differences in expression between CD56^bright^ and CD56^dim^ cells analysed by two Affymetrix microarray platforms: HTA2.0^21^ (black bars) and HG-U133 A/B^22^ (grey bars). Three samples of each subset were analysed on each microarray, for a total of 6 independent sample pairs. Transcription factors showing ≥2-fold difference on both array platforms are shown, ordered by mean fold change. Genes labelled in purple italics have been shown to affect development of ILC and/or NK in mouse models (see references in text). (**B**) Quantitative real-time RT-PCR validation of several differences in transcription factor expression (including some that showed ≥2-fold change in only one microarray dataset). RNA was isolated from human blood CD56^bright^ CD16^−^ cells, CD56^dim^ CD16^+^ cells, and the “intermediate population” displaying CD56^bright^ CD16^+^ phenotype. Transcript levels are quantitated relative to *ACTB* in samples from three individuals (represented by triangle, circle and square symbols) with a bar depicting the geometric mean level of expression. Samples were distinct from those analysed by microarray. *p < 0.05, **p < 0.01, ***p < 0.001, by Tukey’s Multiple Comparison Test after one way ANOVA of Log_10_ transformed values. Trends were consistent if data was normalized to *GAPDH* transcript levels instead of *ACTB*.

Microarray analysis of transcription factors differing between human blood CD56^bright^ and CD56^dim^ populations is shown in Fig. 4A. This list includes differences verified on both microarray platforms with independent samples. CD56^bright^ cells showed higher levels of transcript for *SCML1, RUNX2, SOX4, ZEB1, TCF7, HOXA5, AHR, AFF3, MYC, SSBP2, TCF4*, and *GATA3*. On the other hand, CD56^dim^ cells had higher signal for *IKZF3* (AIOLOS), *MYBL1, RORA, PRDM1* (BLIMP1), *PYHIN1, NFIL3* (E4BP4), *ZEB2, HIPK2, ADRB2, BNC2, BCL11B, TBL1X, CRY1, TCF7L2*, and *TBX21* (T-bet). The differences in a subset of transcription factors were additionally validated by real-time RT-PCR (Fig. 4B), in all cases confirming or trending with the microarray results. “Intermediate” cells with a CD56^bright^ CD16^+^ phenotype were very similar in transcription factor expression to CD56^bright^ CD16^−^ cells (Fig. 4B), as observed previously for cell surface markers^7^.

## Discussion

ImmGen studies have previously defined signature genes that are preferentially expressed in mouse ILC or NK cell subsets^23^. When these signatures were examined in microarray data comparing human peripheral blood subsets, several interesting patterns emerged. The mouse *Core ILC* signature was enriched in human CD56^bright^ cells (Fig. 1A); however, this was driven largely by transcript levels of only a few genes (*IL7R, GPR97*, and *IL2RA*). More in depth comparisons revealed a small subset of genes that are expressed at higher levels in both human CD56^bright^ cells and mouse ILC1 defined by different markers or reporters. This included *IL7R* (CD127), *TNFSF10* (TRAIL), *KIT* (CD117), *IL2RA* (CD25), CD27, *CXCR3, DPP4* (CD26), *GRP183*, *AHR, XCL1*, and MHC class II; most of which were also shown to be preferentially expressed at the cell surface of CD56^bright^ cells (Fig. 2). On the other hand, the mouse *Core NK* signature was enriched in CD56^dim^ cells (Fig. 1B). Human CD56^dim^ and mouse NK cells shared higher expression of *KLRG1, PRF1* (perforin), *GZMA* (granzyme A), *S1PR5, CMKLR1* (chemerin receptor 23), and other genes (Fig. 2A). Interpreted in reverse, this indicates that both human CD56^bright^ and mouse ILC1 have lower levels of these NK cell-associated transcripts.

These similarities could indicate a developmental or lineage relationship between human blood CD56^bright^ cells and mouse CD127^+^ NK1.1^+^ NKp46^+^ ILC. This may imply that human CD56^bright^ cells are ILC-like, or that cells defined primarily by CD127 expression in mouse spleen (with NK1.1 and NKp46) could be analogous to human CD56^bright^ cells rather than *bona fide* ILC1. As suggested recently, NK cells and ILC1 may represent two extremes of a broad spectrum of cells comprising the group-1 ILC population^36^. Human CD56^bright^ cells may inhabit the middle of this spectrum. Strikingly, recent data from rhesus macaques showed that subsets similar to human CD56^bright^ and CD56^dim^ cells predominantly arose from distinct progenitors, with limited overlap and little evidence of differentiation from one subset to the other^8^. It is possible that human blood CD127^+^ CD56^bright^ cells develop from IL-22-producing ILC3 described to exhibit a “stage 3” progenitor phenotype (Lin^−^ CD117^+^ CD161^+^ IL-1R1^+^ CD94^−^)^37–39^, whereas CD56^dim^ NK cells may develop independently from distinct NK cell progenitors (such as those described in ref. 40). Future studies will be required to delineate their lineage relationships (including those with human liver ILC/NK cell subsets^41, 42^).

Alternatively, the subset of genes with common expression in CD56^bright^ cells and mouse ILC may be the result of a similar module of gene regulation, due to actions of particular transcription factors. Strikingly, many transcription factors that differ between CD56^bright^ and CD56^dim^ human blood populations have been shown to affect ILC development and function in mouse knockout models. CD56^bright^ cells show higher levels of *GATA3*, *AHR, TCF7*, and *RUNX2* transcripts (Fig. 4A). Upon gene disruption in mouse hematopoietic cells, *Gata3* was shown to be necessary for development of ILC3, ILC2, and CD127^+^ ILC1, while CD127^−^ NK cells (and DX5^−^ liver ILC) were largely maintained^15, 43^. Similarly, mice deficient in *Ahr* displayed largely intact NK cell populations, despite deficiencies in ILC3 subsets^44–46^ and liver ILC1^47^. *Tcf7* (encoding TCF-1 protein) affects all mouse ILC/NK lineages and is expressed in early ILC/NK progenitors^48, 49^. Among the Runx family of transcription factors, *Runx3* impacts mouse ILC3, ILC1, and NK development^50^. Interestingly, *Tcf7* ^GFP^ reporter mice and mice with YFP inserted at the distal *Runx3* promoter both appear to show higher levels of fluorescence in ILC1 and ILC3 versus NK cells^48, 50^. Human *RUNX3* transcripts levels were high in both CD56^bright^ and CD56^dim^ subsets (not shown) while CD56^bright^ cells selectively expressed greater *RUNX2* mRNA (Fig. 4A,B).

On the other hand, human CD56^dim^ cells show higher levels of mRNA for *RORA*, *BCL11B*, and *TBX21* (Fig. 4A), which have also been implicated in ILC development. *Rora* ^−/−^ and *Bcl11b* ^*−*/−^ mice have severe deficiencies in ILC2^51–55^. Mice deficient in *Tbx21* (T-bet) lack NK1.1^+^ NKp46^+^ TRAIL^+^ DX5^−^ ILC1 cells in liver, while DX5^+^ NK cells are partially retained but phenotypically altered^12, 17, 18^. In summary, the transcription factors that may control the differentiation of human CD56^bright^ and CD56^dim^ cells appear to be closely related with those that affect ILC versus NK cell differentiation in the mouse. One exception is Eomes, implicated in mouse studies as critical in the regulation and demarcation of NK cell versus ILC1 development^12, 17, 18^. *EOMES* did not score as significantly different between the human subsets in microarray. Both CD56^bright^ and CD56^dim^ cells showed high levels of EOMES protein by intracellular flow cytometry (Fig. 1C). Interestingly, the subset of human liver ILC/NK that express the characteristic mouse ILC gene CXCR6 are also Eomes^high^, suggesting differences between species^41^.

The CD56^dim^ population also possesses higher levels of several other transcription factors associated with the formation or function of mature mouse NK cells, including *NFIL3* (E4BP4), *PRDM1* (BLIMP1), and *IKZF3* (AIOLOS) (Fig. 4A). *Nfil3* ^*−/−*^ mice lack most NK cells^56, 57^, but *Nfil3* is also necessary for the formation of ILC progenitors and most mouse ILC lineages with the apparent exception of liver DX5^−^ ILC1^15, 58–61^ *. Prdm1* deficiency has been shown to affect the proliferation and maturation phenotype of mouse DX5^+^ NK1.1^+^ NK cells, exhibiting increased CD27, CD117 and decreased KLRG1^62^, molecules that are also differentially expressed between human CD56^bright^/CD56^dim^ and mouse ILC1/NK cell subsets (Fig. 1,2). Selective expression of *PRDM1* in human CD56^dim^ cells has been additionally confirmed^62, 63^. *Ikzf3* has also been shown to affect mouse DX5^+^ NK1.1^+^ NK cell maturation markers and function^64^.

Interestingly, human CD56^dim^ cells express higher levels of transcripts for *ZEB2*, while CD56^bright^ cells show greater amounts of message for the related molecule, *ZEB1* (Fig. 4A,B). *Zeb2* was also selectively expressed in mouse NK cells in comparisons versus ILC1 in all three tissues examined (Fig. 2, Suppl. Figure 3,4), and is included in the ImmGen mouse *Core NK* signature^23^. Targeted deletion of *Zeb2* resulted in impaired NK cell maturation affecting several molecules observed to differ between CD56^bright^ and CD56^dim^ subsets (including CD27, KLRG1, S1PR5, and CXCR3) in a gene-dosage dependent manner^65^. It is intriguing to speculate that the ZEB1/ZEB2 balance could form a molecular switch controlling the differentiation fate of ILC/NK subsets. It is also striking that *ZEB2, TBX21, PRDM1*, and *IKZF3*, each expressed at higher levels in human CD56^dim^ cells, all show some commonalities in mouse knockout models^62, 64, 65^.

Many of the transcription factors that differ between CD56^bright^ and CD56^dim^ cells also play roles in T cell development and differentiation. For example, *Gata3*, *Tcf7*, *Lef1*, *Bcl11b* (and *Runx1*/*Runx3)* were included in a regulatory network controlling mouse thymic T cell development, which also includes *Kit* (CD117) and *IL7r* (CD127)^66^. Similarly, *Sox4*, *Tcf7*, *Prdm1*, *Runx2*, *Gata3*, and *Tbx21* (along with *Eomes*) are part of a bioinformatically determined transcription factor network associated with mouse T cell memory formation^67^. Furthermore, perturbation analyses performed by retroviral transduction of each gene into T cells has suggested complex regulatory effects amongst these transcription factors^67^. For example, overexpression of Sox4 downregulated mRNA for *Tcf7*, *Eomes*, *Runx2*, and *Tbx21*, while overexpression of Tcf7 downregulated *Sox4*, *Eomes*, and *Prdm1* transcripts; in addition, transduction of Prdm1 repressed transcription of both *Sox4* and *Tcf7* ^67^. It is likely that a similar network of interactions (including additional factors from Fig. 4) governs differentiation into human blood CD56^bright^ and CD56^dim^ subsets. Future studies will determine if this network is analogous to that regulating ILC versus NK cell differentiation in the mouse, which in turn may explain the cross-species transcriptome similarities observed (Figs 1, 2 and 3).

## Methods

### Microarray data analyses

Affymetrix HTA2.0 chip CEL files from flow cytometry sorted human peripheral blood CD56^bright^ (CD3^−^ CD56^bright^ CD16^−^) and CD56^dim^ (CD3^−^ CD56^dim^ CD16^+^) subsets (from ref. 21) (NCBI GEO accession numbers GSM2278891-GSM2278896 inclusively) were normalized by robust multi-array average (RMA) algorithm using Expression Console version 1.4.1.46 with “Gene level RMA sketch” settings, and annotated with Affymetrix release 35.1 transcript cluster annotations. Unless otherwise noted, the shown fold change values are calculated from this normalized HTA2.0 data. HG-U133A/B CEL files from three additional independent samples of each of human blood CD56^bright^ and CD56^dim^ cells (from ref. 22) (GSM2108750- GSM2108755 inclusively and GSM2108760-GSM2108765 inclusively) were normalized using Partek Genomics Suite 6.6 using the RMA algorithm with default settings and annotated with Affymetrix annotations (release 35).

CEL files encoding microarray data describing mouse spleen, liver, and small intestine ILC subsets from the ImmGen project^23^ were downloaded from NCBI GEO with accessions GSM1585312-GSM1585337 inclusively, then RMA normalized with Partek Genomics Suite 6.6 (with default settings) and annotated with Affymetrix release 35 transcript cluster annotations. Additional CEL files analysing small intestine ILC and NK populations were also downloaded from ArrayExpress (E-MTAB-2428)^12^ and similarly analysed using Partek Genomics suite. Human transcription factors were identified using a list compiled from three sources (see refs 68–70).

**Gene Set Enrichment Analysis** was performed with GSEA v2.2.0 or v2.2.3 (Broad Institute)^24^ using default settings with the exception of gene set randomization as the permutation type, due to the limited number of phenotypes analysed.

### Cross-species transcriptome comparisons

Single microarray probesets were chosen to represent each gene, prioritizing: lack of cross reactivity, recognition of coding regions, and higher fold changes between subsets, when such information was annotated. Mouse and human gene expression results were synchronised using NCBI Homologene database (release 68) allowing examination of 16,351 mouse-human homologous gene pairs. Spearman correlations between human and mouse relative gene expression were performed on log_2_ transformed fold change values, examining only genes for which expression differed by more than two fold between the mouse subsets. Lists of signature mouse transcripts were also converted to human homologues via NCBI Homologene.

### Human blood samples

Peripheral blood immune cell subsets were isolated from de-identified blood bank buffy-coat or leukopack samples. Depending on the site of the experiments and source of samples (i) NIH Office of Human Subjects Research Protections, and (ii) the Committee on the Use of Human Subjects (the Harvard institutional review board) determined that this use of this material is exempt from the requirements of IRB review, therefore not requiring written consent.

### Quantitative Real-time RT-PCR

Leucopacks were treated using a negative enrichment antibody mixture (NK RosetteSep, Stem Cell Technologies). Enriched cells were then flow cytometry sorted in order to isolate CD56^bright^ (CD3^−^ CD56^bright^ CD16^−^), CD56^dim^ (CD3^−^ CD56^dim^ CD16^+^) subsets, as well as an intermediate population which is CD3^−^ CD56^bright^ CD16^low^. Cells were washed 3-4 times with PBS and cell pellets stored at -80C. RNA was isolated using the RNeasy Plus Mini Kit (Qiagen). All isolated RNAs showed an RNA Integrity Number of >9.3 when tested by Agilent Bioanalyzer. First strand cDNA was synthesized from 100-200ng total RNA using RT^2^ First Strand Kit (Qiagen) and PCR reactions were performed with RT^2^ SYBR Green Mastermix (Qiagen) in triplicate. The PCR reaction profile consisted of 10 min at 95 °C, followed by 40 cycles of 15 s at 95 °C, and 1 min at 60 °C in BioRad CFX96 or Stratagene MX3000p thermocyclers. Primers were derived from custom designed RT^2^ Profiler PCR Arrays (SuperArray/Qiagen). Samples with multiple melting-curve peaks were not included in analyses.

### Flow cytometry

After overnight at room temperature, NK cells were isolated from human blood buffy-coat samples with Rosettesep as above, and stained with the following mAb clones recognizing CD56 (NCAM16.2), CD16 (3G8), CD127 (HIL-7R-M21), HLA-DR (G46-6), CD25 (M-A251), TRAIL (RIK-2) from BD Biosciences, CD3 (OKT3), CD127 (A019D5), CD27 (M-T271), GPR183 (SA313E4), CD26 (BA5b), CXCR3 (G025H7), or CD117 (104D2) from BioLegend. Staining for EOMES was performed with clone WD1928 and transcription factor staining buffer set (both from Affymetrix EBioscience).

## Electronic supplementary material

Supplemental Figures
