# Supplementary material for: Transcriptome analysis reveals similarities between human blood CD3− CD56bright cells and mouse CD127+ innate lymphoid cells
Source: Sci Rep. 2017 Jun 14;7:3501. doi: 10.1038/s41598-017-03256-0 (PMC5471261; doi:10.1038/s41598-017-03256-0)
Supplement: Supplementary file 1 — Supplemental Figures [file 41598_2017_3256_MOESM1_ESM.pdf]

Supplemental Figures

Transcriptome analysis reveals similarities between human blood CD3$^{–}$ CD56^{bright} cells and mouse CD127^{+} innate lymphoid cells

David S.J. Allan, Ana Sofia Cerdeira, Anuisa Ranjan, Christina L. Kirkham, Oscar A. Aguilar, Miho Tanaka, Richard W. Childs, Cynthia E. Dunbar, Jack L. Strominger, Hernan D. Kopcow, and James R. Carlyle

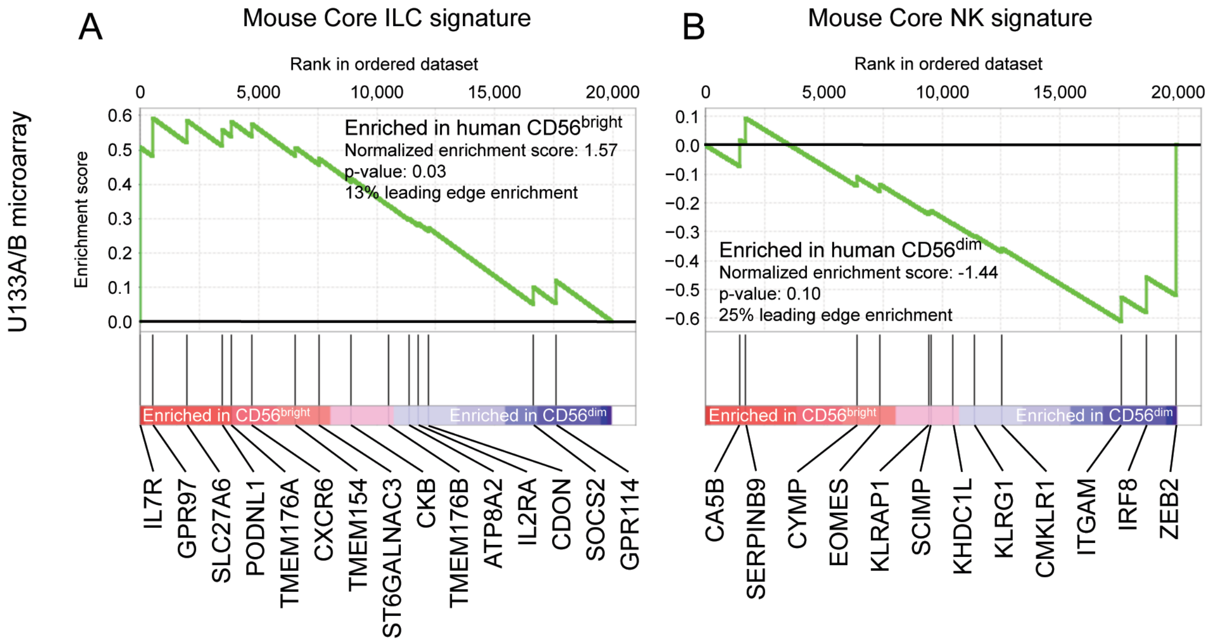

Supplemental Figure 1. Second dataset: Signature genes, higher in multiple mouse ILC subsets, are enriched in human blood CD3$^{–}$ CD56^{bright} cells, while mouse NK cell signature genes are enriched in human CD3$^{–}$ CD56^{dim} cells. Analysis, as described in **Figure 1**, but using microarray data from the HG-U133A/B platform acquired with independent samples of human blood CD3$^{–}$ CD56^{bright} CD16$^{–}$ and CD3$^{–}$ CD56^{dim} CD16^{+} populations[22]. Three samples of each subset were used for analysis.

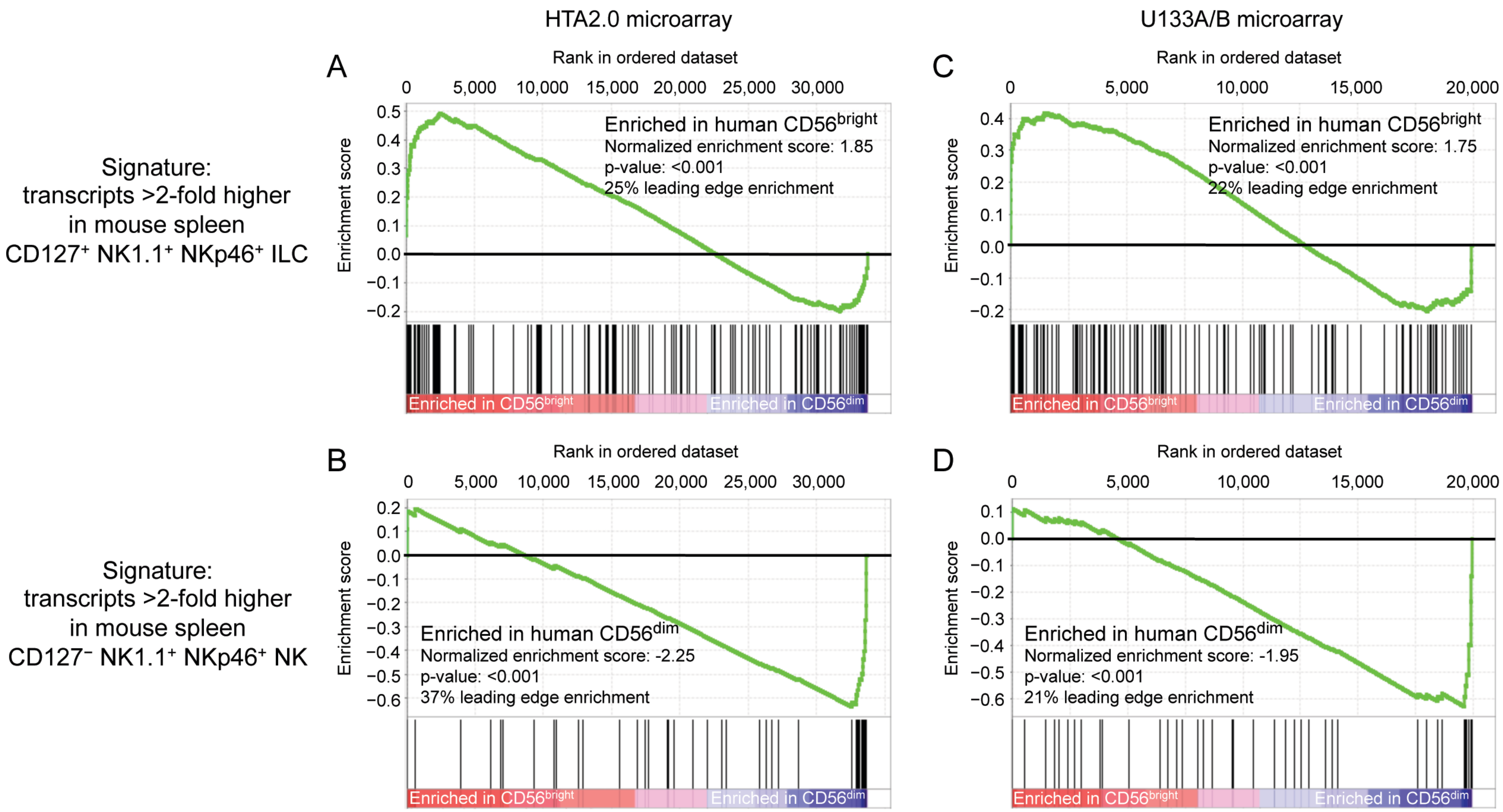

Supplemental Figure 2. Transcripts higher in mouse spleen CD127⁺ NK1.1⁺ NKp46⁺ ILC1 are enriched in human blood CD56^{bright} cells, while transcripts higher in mouse CD127⁻ NK1.1⁺ NKp46⁺ NK cells are enriched in human CD56^{dim} cells. Using ImmGen data from reference 23, lists of transcripts ≥2-fold higher in either mouse subset were created (corresponding with transcripts outside the vertical lines in **Figure 2A**). These lists were used for GSEA analyses with microarray data contrasting human $CD3^{-}$ $CD56^{bright}$ $CD16^{-}$ and $CD3^{-}$ $CD56^{dim}$ $CD16^{+}$ populations on either (**A,B**) HTA2.0[21] or (**C,D**) HG-U133A/B[22] platforms.

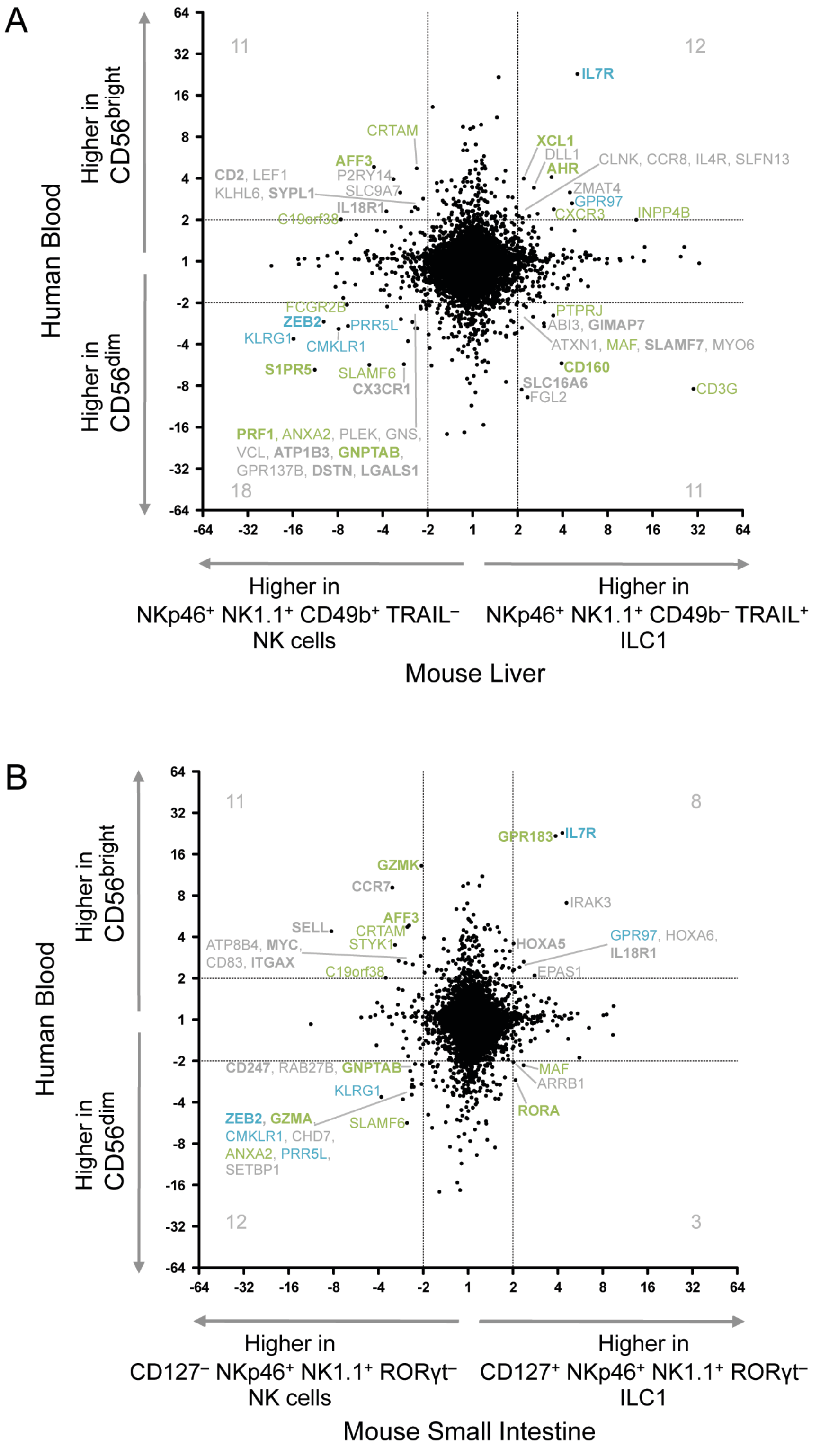

Supplemental Figure 3. Comparison of expression differences between human blood CD56^{bright} and CD56^{dim} cells with those observed between mouse ILC1 and NK cells in several tissues reveals several genes showing similar patterns. Analyses, as described in **Figure 2A**, comparing expression differences between human CD56^{bright} and CD56^{dim} cells with differences between **(A)** mouse liver ILC1 and NK cells, or **(B)** mouse intestinal ILC1 and NK cells. Human subset data from HTA2.0 microarray[21]. Mouse data from reference 23.

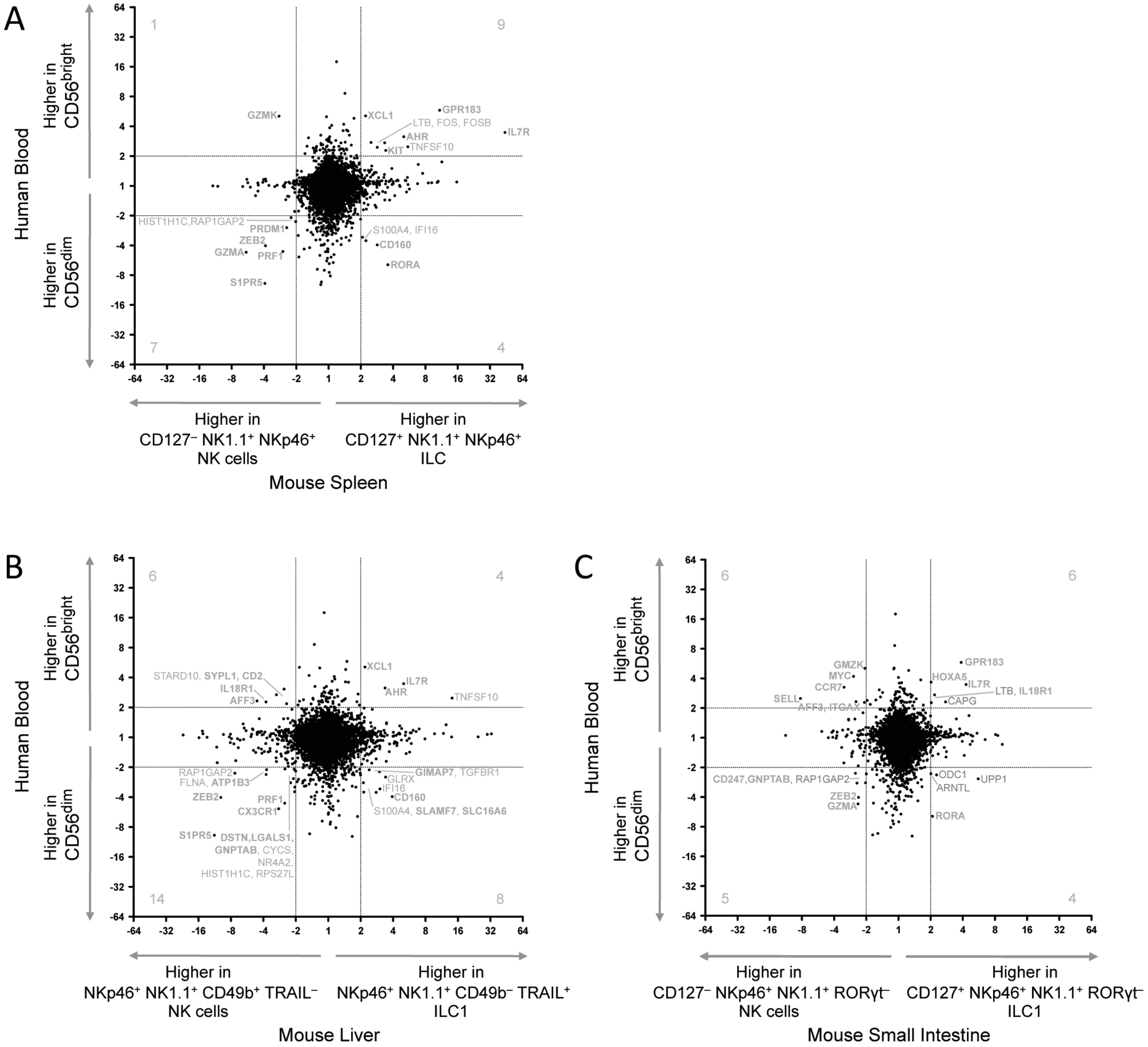

Supplemental Figure 4. Second dataset: Expression differences between mouse spleen CD127⁺ NK1.1⁺ NKp46⁺ ILC and CD127⁻ NK1.1⁺ NKp46⁺ NK cells show commonalties with differences between human blood CD56^{bright} and CD56^{dim} cells. Analyses, as described in **Figure 2A** and **Suppl. Figure 3**, but using independent human microarray data from the HG-U133A/B platform[22]. Considering only the subset of transcripts differing ≥2-fold between mouse subsets, results in (**A**) showed Spearman correlation of r=0.31 (p<0.0001) while (**B,C**) were not significant. Comparing transcript counts ≥2-fold higher in *both* human and mouse subsets (enumerated in each corner) by Fisher's exact test showed (**A**) p=0.02 while (**B,C**) were not significant.

U133A/B microarray

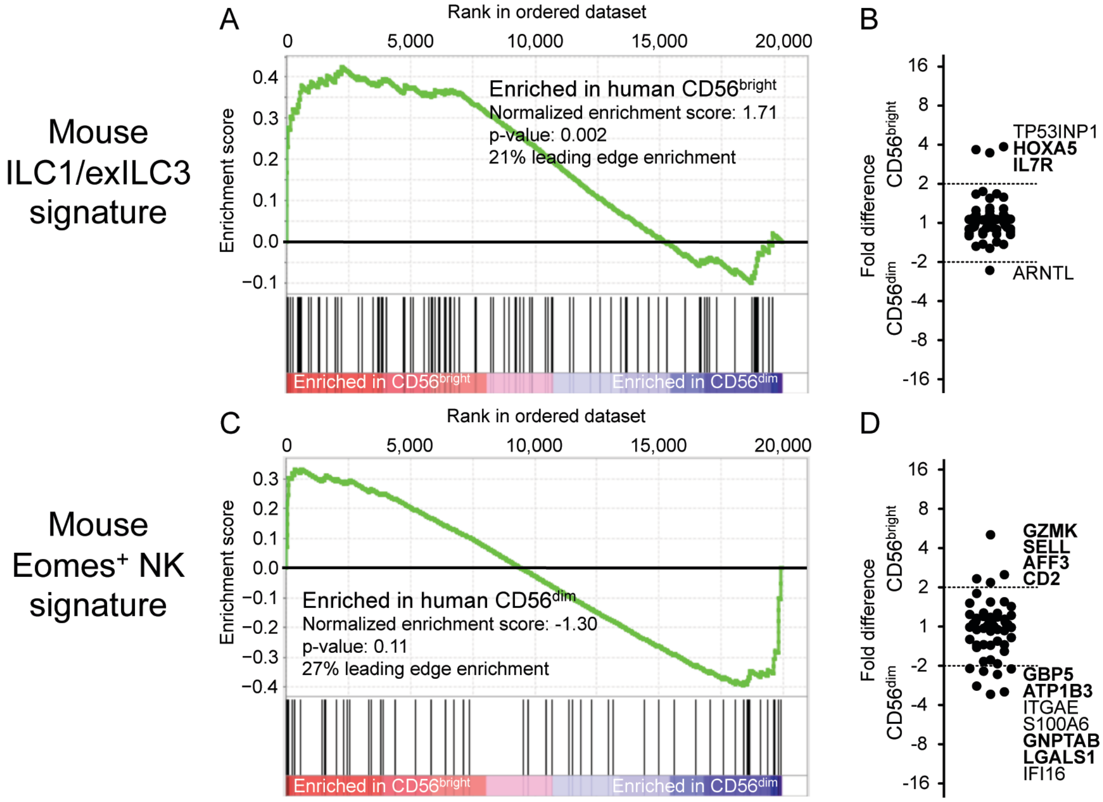

Supplemental Figure 5. Second dataset: Transcripts with higher expression in both mouse ILC1 and ex-ILC3 subsets show enrichment in human CD56^{bright} cells. Signature enrichment was examined by GSEA as described in **Figure 3**, but using human microarray data from HG-U133A/B platform comparing CD56^{bright} and CD56^{dim} cells[22].

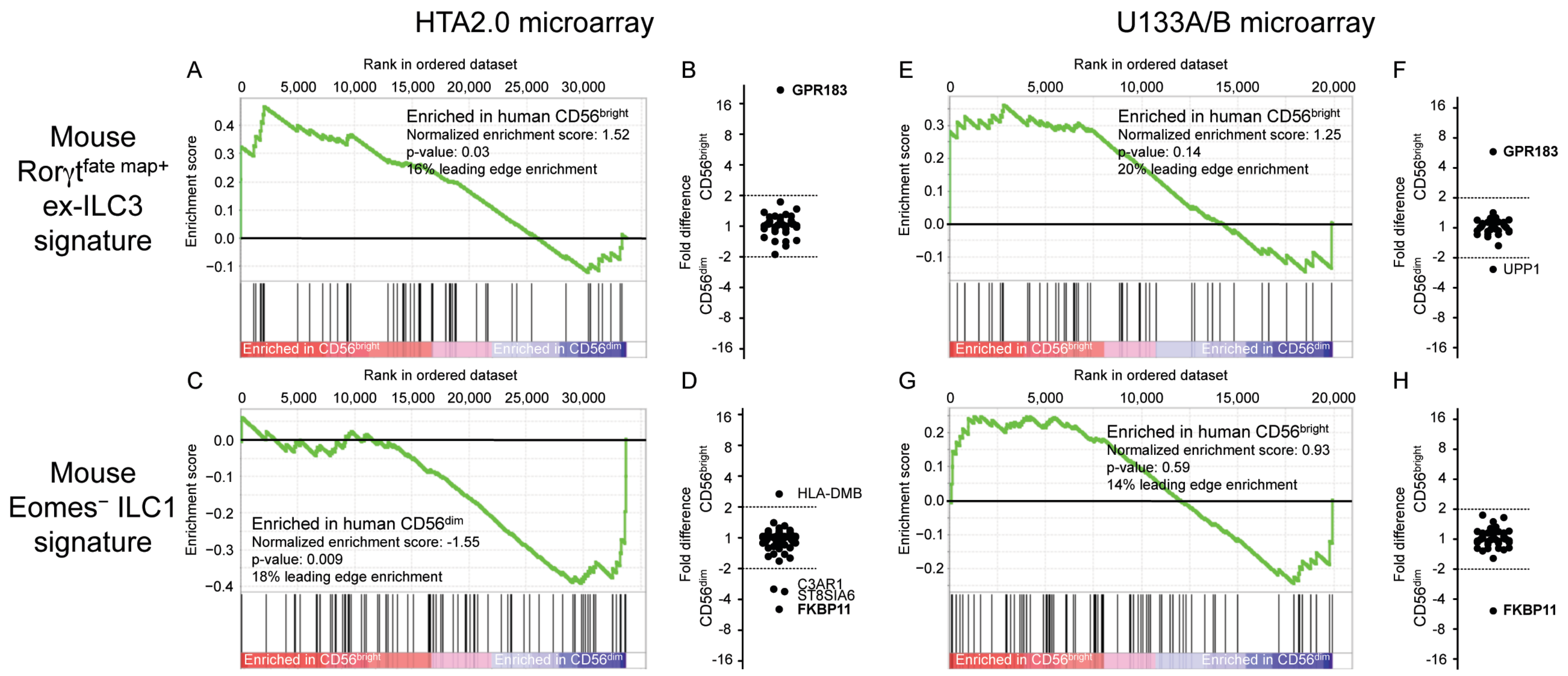

Supplemental Figure 6. With the exception of *GPR183*, signature transcripts of either mouse ex-ILC3 or ILC1 subsets are not clearly enriched in human CD56^{bright} or CD56^{dim} subsets. GSEA was performed as described in **Figure 3**, using human microarray data from both **(A-D)** HTA2.0[21] and **(E-H)** HG-U133A/B[22] platforms. Signatures were as follows: **(A,B,E,F)** *Mouse Ror$\gamma t^{fate\text{-}map+}$ ex-ILC3 cell signature* (genes with ≥2-fold higher expression in *both* ex-ILC3 versus ILC1 and ex-ILC3 versus NK comparisons); and **(C,D,G,H)** *Mouse Eomes⁻ ILC1 cell signature* (genes with ≥2-fold higher expression in *both* ILC1 versus ex-ILC3 and ILC1 versus NK comparisons) generated from data in reference 12.

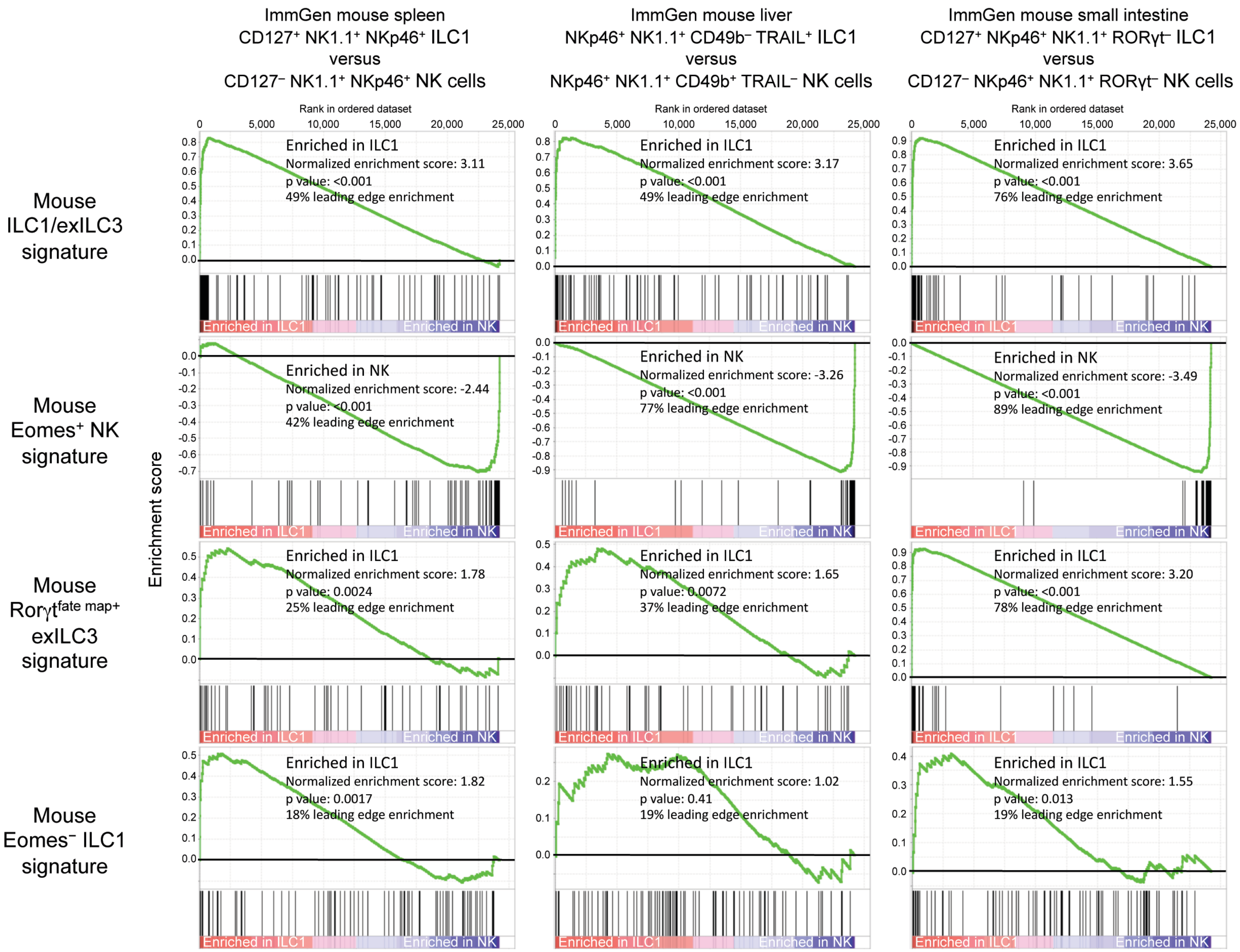

Supplemental Figure 7. Mouse intraspecies comparison of ILC subset signatures. Mouse ImmGen microarray comparisons of cell populations indicated (from reference 23) were queried by GSEA using signatures used previously in **Figure 3** and **Suppl. Figures 5,6** (compiled from mouse microarray data describing ILC subsets (from reference 12).
